# Correlates of public support toward federal funding for harm reduction strategies

**DOI:** 10.1186/s13011-015-0022-5

**Published:** 2015-06-30

**Authors:** Magdalena Kulesza, Bethany A. Teachman, Alexandra J. Werntz, Melissa L. Gasser, Kristen P. Lindgren

**Affiliations:** Department of Psychiatry, University of Washington, 1100 NE 45th Street, Seattle, WA 98105 USA; RAND Corporation, 1776 Main St, Santa Monica, CA 90407 USA; University of Virginia, Department of Psychology, 102 Gilmer Hall, Charlottesville, VA 22904 USA

**Keywords:** Public stigma, Intravenous drug use, Harm reduction, Safe injection facilities, Needle and syringe programs

## Abstract

**Background:**

Historically, US federal policy has not supported harm reduction interventions, such as safe injection facilities (SIFs) and needle and syringe programs (NSPs), which can reduce the burden associated with injection drug use. Given recent increases in abuse of both legal and illegal opioids, there has been a renewed debate about effective ways to address this problem. The current study (1) assessed participants’ support for SIFs and NSPs, and (2) evaluated several demographic factors (e.g., age, gender, race, education, political ideology, and religiosity) and individual differences in stigmatizing beliefs about people who inject drugs (PWID) that might relate to support for these interventions.

**Methods:**

U.S. adults (*N* = 899) completed a web-based study that assessed self-reported support for NSPs and SIFs, and stigma about PWID.

**Results:**

The majority of participants were at least somewhat supportive of both NSPs and SIFs. Regression analyses indicated greater support for NSPs and SIFs was predicted by more liberal political ideology, more agreement that PWID deserve help rather than punishment, older age, and male gender. Also, participants who endorsed lower stigma about PWID were more supportive of NSPs and SIFs. Race, religiosity, and education did not predict support for NSPs and SIFs.

**Conclusions:**

Most participants tended to report support for harm reduction strategies. Age, political ideology, and individual differences in stigmatizing beliefs about PWID were significantly associated with support. Given the potential malleability of stigmatizing beliefs, efforts that seek to shift stigma about PWID could have important implications for public policy towards harm reduction strategies for PWID.

## Background

Injection drug use is associated with significant societal costs, and both opiate use and related deaths are increasing nationwide [5]. Although harm reduction strategies, such as needle and syringe programs (NSPs) and safe injection facilities (SIFs), have been shown to be effective at reducing negative consequences of drug use [23, 41, 42], these strategies are not currently part of federal drug control policy in the U.S., likely due to political, legal, and moral objections [1, 35], as well as stigma, and perception of drug use as a criminal behavior, rather than a health problem [33]. Still, a number of U.S. states provide funding for some form of harm reduction interventions, and there is renewed debate about the most effective strategies to address the burden of opiate addiction, and injection drug use [31]. Given the significant impact of public opinion on policy decisions [4], it is crucial to gain a better understanding of attitudes toward harm reduction policies held by the individuals in the U.S. While previous public opinion polls have contributed to our knowledge, they have tended to look at attitudes on average, rather than considering individual differences in support for harm reduction strategies [40]. Thus, the goal of the current study was to evaluate several demographic factors and individual differences in beliefs that might differentially predict support for NSPs and SIFs.

We selected NSPs due to their controversial status in the U.S. in spite of data supporting their efficacy in reducing harm among PWID [41, 42]. Our interest in SIFs was motivated by two reasons. First, there is accumulating research support for their efficacy in reducing high risk behaviors while increasing healthcare participation among PWID [24, 43, 44]. Second, to our knowledge, U.S. adults’ support for SIFs has not been evaluated. NSPs refer to the provision of sterile injection equipment to PWID to reduce transmission of infectious disease [18, 20], whereas SIFs refer to locales where PWID can more safely (i.e., supervised by a nurse or social worker) consume drugs. There is robust support for NSPs efficacy in reducing the transmission of HIV without increasing drug use [35, 41, 42]. While NSPs have a longer history and more scientific scrutiny, SIFs have received increased attention from researchers in recent years. SIFs were established to reduce risk of overdose, infectious disease transmission, and negative impact on public order [2, 17]. A growing body of research indicates that SIFs reduce high-risk behaviors among PWID, such as syringe sharing [16, 21, 22, 24, 44], while facilitating provision of health care and addiction treatment [14, 38] without increasing drug use or injecting behavior [23, 25, 43].

In contrast to the lack of data about SIFs in the U.S., there are some data describing support towards NSPs. Specifically, a systematic analysis of national public opinion polls evaluating support towards NSPs indicated that, depending on the poll, between 29 and 66 % of individuals endorsed favorable views [40]. These statistics represent quite a wide range of support, and the authors suggested several reasons for the variability [40]. First, it might be partially due to how the survey questions are worded, such as the use of loaded terms like “drug addicts’ or “junkies,” that could contribute to more bias-driven responses. In fact, the authors documented that simply replacing “those addicted to illegal drugs” with “drug addicts” was related to a 9 % drop in support towards NSPs. Hence, the authors recommended avoiding pejorative terms in survey questions. Second, they identified the beliefs and/or bias of an organization as a potential explanatory factor. Given that polls sponsored by more liberal organizations reported more support towards NSPs than those sponsored by more conservative organizations, the authors recommended that polls should be sponsored by organizations without a public position on the issue. Third, the lack of knowledge about the scientific evidence supporting efficacy of these strategies in reducing HIV infection may be another reason for the inconsistent findings and low support. Last, the authors noted the importance of evaluating factors that might influence public support toward harm reduction strategies; the focus of the current study.

We are not aware of any published research that directly assesses predictors of public support for harm reduction services in the US. When considering plausible candidates, U.S. and international research related to other drug policies is informative. First, stigma toward individuals coping with substance abuse is one possible factor; here, we focus on public stigma, which is the endorsement of negative stereotypes by society at large, which can then lead to negative emotional reactions (e.g., anger, fear) and discrimination toward individuals belonging to the stigmatized group [10]. Given stigma relates to a preference for more punitive, rather than help-related, responses to individuals with substance abuse [26, 28, 33], it is expected that greater stigma will predict rejection of harm reduction approaches in the current study.

Second, findings from two studies conducted in Canada assessing participants’ views about harm reduction programs indicated that several factors were associated with greater support: higher income and education [13, 39], younger age [39], personal use of cocaine or marijuana in the past year [13], favorable view of marijuana decriminalization [13], and perception of individuals with substance abuse as ill people [13]. Gender, however, was not a significant predictor in one study [13] while another reported that women were more supportive [39]. Also, a study evaluating U.S. public support for increased government spending for “dealing with drug addiction” found that while all demographic groups were generally supportive of increased spending, after controlling for general domestic spending, men (vs. women), conservatives (vs. liberals), older (vs. younger) individuals, and residents of the South (vs. North) were more supportive of spending [32]. These surprising results were speculated to stem from participants’ assuming that drug-problem spending would be more punitive/criminal justice-based, rather than public health-oriented. Interestingly, race/ethnicity was not a significant predictor. Because these data were either from Canada [13, 39] or are dated [32], we evaluated predictors of current support toward harm reduction services in a large U.S. sample.

### Overview

We sought to add to the literature in the following ways. First, evidence suggests that even after accounting for other factors such as the influence of interest groups, public opinion has a significant impact on public policy [4]. Hence, by evaluating public views towards provision of federal funding for harm reduction strategies, we increase our knowledge about a potential source of the current U.S. federal ban on support for harm reduction strategies. Second, we addressed some of the criticisms of the extant literature [40] by avoiding use of prejudicial terms in our survey, framing questions neutrally, describing PWID in behavioral terms, and linking the use of harm reduction strategies to scientific evidence. Third, given the dearth of research looking at correlates of U.S. public opinion toward government funding for harm reduction services, we also assessed whether public stigma toward PWID and a series of demographic factors were related to the extent of support. Note, we are not attempting to conduct an epidemiological study among a representative sample; rather, to identify promising predictors. We used a sample that was relatively diverse and large enough to enable analyses of various individual differences, but we make no claims that the sample is representative. Finally, we are the first to assess support toward SIFs among U.S. adults.

Our aims, therefore, were to: (1) assess participants’ opinions about providing government funding for SIFs and NSPs, and (2) evaluate several demographic and individual difference factors (age, gender, race, education, political ideology, religiosity, and public stigma towards PWID) that might relate to support for these strategies.

## Methods

### Participants

Participants were 899 volunteers at the Project Implicit research website (https://implicit.harvard.edu/implicit/) between July 24, 2013 and August 19, 2013. Out of 1453 individuals randomly assigned to this study, 1157 consented to participate, and of those, 899 individuals completed study procedures and materials presented in this manuscript. There were no significant differences on demographic variables between study completers and non-completers.

Project Implicit is a nonprofit website that investigates attitudes and beliefs that are relatively outside of conscious control. Individuals are randomly assigned to study topics after registering on the site and agreeing to participate in a study. Only individuals over 18, fluent in English, and citizens of the U.S. were eligible for the present study; 98.3 % were current residents of the U.S. Project Implicit samples are relatively diverse, and there is evidence supporting the validity of Project Implicit’s approach to web-based data collection [29, 30]. Participants are volunteers and are not compensated for study participation. Volunteers find the website through a variety of sources, such as surfing the web, an assignment for school or work, or through direct searches. See Table 1 for sample characteristics.

**Table 1:** Study participant characteristics (*n* = 899)

Gender (% women)	62.4
Age (*M, SD,* range)	38.97 (13.65, 19–90)
Ethnicity (%)	
Hispanic or Latino	7.3
Not Hispanic or Latino	79.5
Unknown or did not report	13.2
Race (%)	
Caucasian	69.9
Black or African American	13.2
More than one race	7.3
Asian	4.2
Other or unknown	3.1
American Indian or Alaskan Native	.7
Native Hawaiian or other Pacific Islander	.7
Did not report race	.9
Education (%)	
Less than a high school degree	1.4
High school degree, some college, or an Associate’s degree	39.2
Bachelor’s degree or some graduate school	28.1
Advanced degree (e.g., PhD, MD)	30.4
Did not report	.9
Political ideology (%)	
Strongly liberal	18.5
Moderately liberal	23.5
Slightly liberal	8.3
Moderate or neutral	29.1
Slightly conservative	6.7
Moderately conservative	7.1
Strongly conservative	2.7
Did not report	4.1
Religiosity (%)	
Not at all religious	36.4
Somewhat religious	26.9
Moderately religious	23.1
Very religious	10.3

### Measures

#### Public policy

To assess endorsement of NSPs, participants rated agreement with the statement: “Federal funds should be made available for needle/syringe exchange programs if it can be shown that they reduce the transmission of HIV among users and do not encourage the use of illegal drugs.” Similarly, to assess endorsement of SIFs, participants rated agreement with the statement: “Supervised injection facilities for current intravenous drug users (i.e., legally sanctioned and medically supervised facilities to consume drugs) should be made available through federal funds if it can be shown that they reduce overdose deaths or infectious disease among users.” Both items were rated on a four-point scale (1 = *strongly disagree* to 4 = *strongly agree*). These questions were adapted from previous work examining public opinion concerning SIFs [13].

#### Public stigma

The Social Distance Scale [27], an instrument assessing attitudes towards interacting with someone with mental illness, was adapted for the current study. All seven items (e.g., “How would you feel about renting a room to a former mental patient?”) were retained in the current study; however, each was modified to reference PWID instead of a “former mental patient.” Participants rated each of the seven items on a four-point scale (1 = *definitely willing to* 4 = *definitely unwilling to*), with higher scores corresponding to greater desire for social distance. Cronbach’s alpha in the current sample was .83, and the scale has adequate psychometric properties when modified for individuals coping with substance use-related problems [3].

The Perceived Dangerousness Scale [27], an instrument developed to assess beliefs about potential dangerousness of individuals coping with mental illness, was modified to assess perceived dangerousness of PWID. Five of the original eight items (e.g., “One important thing about former mental patients is that you cannot tell what they will do from one minute to the next.”) were retained and modified for the current study such that “former mental patient” was replaced to reference PWID instead. Response options to each of the five items were on a six-point scale (1 = *strongly disagree* to *6 = strongly agree*). Higher scores correspond to greater stigma. Cronbach’s alpha in the current sample was .71.

#### Help-punishment differential

To assess individuals’ self-reported support for helping or punishing PWID, participants were asked to indicate their inclination towards helping vs. punishing PWID. Response to this single item measure used a nine-point scale (1 = *extremely deserving of help* to 9 = *extremely deserving of punishment*) with higher scores indicating greater endorsement of punishing PWID.

#### Demographic information

Participants reported gender, birthdate, race, ethnicity, education level, religiosity, and political ideology. Both religiosity and political ideology were assessed using anchored four-point and seven-point Likert Scales respectively. Thus, both variables were entered into the regression analyses as-is. In addition, we asked participants to select the highest level of education they completed from the list of 14 categories. To simplify the analytic approach and make the variable easier to interpret, these categories were collapsed to four more general categories shown in Table 1. Lastly, this variable was treated as an ordinal variable and used in regression analyses as-is. See Table 1 for response options and frequencies.

### Procedure

The current study was approved by the University of Virginia’s Institutional Review Board for Social and Behavioral Sciences. Following random assignment to the present study at the Project Implicit site, participants read the informed consent agreement before beginning the study. After participants completed all questionnaires assigned in random order, they were debriefed on the purpose of the study.

#### Analytic strategy

Given that our key outcome variables (i.e., support for NEPs and SIFs) were continuous and normally distributed, separate linear regression models were used to evaluate the predictors. For each model, all variables were entered simultaneously given there was insufficient rationale to suggest that any set of predictors ought to be evaluated separately in stepwise regression. SPSS software was used for all analytical procedures.

## Results

See Table 2 for descriptive statistics for each candidate variable. The majority of participants were supportive of both NSPs and SIFs. More than 81 % either “somewhat” or “strongly” agreed with funding NSPs, and more than 60 % either “somewhat” or “strongly” agreed with funding SIFs. Support for NSPs was significantly stronger than for SIFs, *t* (765) = 17.02, *p* < .001.

**Table 2:** Descriptive statistics for each candidate variable and correlations with support toward NEPs and SIFs (*n* = 899)

Variable	1	2	M	SD
1. NSPs policy^a^	--		3.29	.93
2. SIFs policy^a^	.53***	--	2.69	1.07
3. Pol. Ideology^b^	.41***	.38***	.87	1.66
4. Gender	−.06	−.05		
5. Age	.15***	.16***	38.97	13.65
6. Race	−.09*	−.00		
7. Education	.22***	.15***		
7. PDS^c^	−.34***	−.27***	3.62	.93
8. SDS^d^	−.26***	−.28***	3.08	.53
9. Help/punishment^e^	−.38***	−.31***	2.39	1.61
10. Religiosity	−.21***	−.17***	2.06	1.02

^a^NSPs and SIFs policy = policy question assessing participants’ support toward allocating government funds to Needle and Syringe Programs (NSPs) and Safe Injection Facilities (SIFs), respectively. ^b^Pol. Ideology = political ideology. ^c^PDS = Perceived Dangerousness Scale. ^d^SDS = Social Distance Scale. ^e^Help/punishment = semantic differential question assessing participants’ view whether IDUs need help vs. punishment. Gender was coded as 0 = men and 1 = women. Also, given that majority of our participants identified as White or Caucasian (69.9 %) we recoded our Race variable to: 0 = White and 1 = everyone else. *p < .05, **p < .01, ***p < .001

Regarding support toward NSPs (see Table 3), the overall regression model was significant and accounted for 30 % of the variance in support for NSPs, *F* (9, 610) = 29.64, *p* < .001. Consistent with predictions, older age, more liberal political ideology, lower perceptions that PWID are dangerous, and stronger belief that PWID deserve help rather than punishment were all positively associated with support. While we did not propose specific directional relationships for gender, race, education, and religiosity, we found that male gender was significantly associated with higher support toward NSPs. However, race, religiosity, and education were not significantly related to support for NSPs. Last, contrary to expectation, desire for distance from PWID was not a significant predictor. Political ideology and beliefs about punishment/help for PWID were the strongest predictors with medium effect sizes. Age, gender, and beliefs about the dangerousness of PWID were significant predictors, but their effect sizes were small.

**Table 3:** Regression Models Predicting Support toward Needle and Syringe Programs (NSPs) and Safe Injection Facilities (SIFs), *n* = 899

	Standardized *B*	SE *B*	*t*	Cohen’s *d*
	Support Toward NSPs			
Gender	−0.09**	0.07	−2.62	0.21
Age	0.12**	0.01	3.47	0.28
Race	−0.06	0.07	−1.76	0.14
Education	0.07	0.03	1.97	0.16
Political Ideology	0.24***	0.02	6.15	0.49
Religiosity	−0.07	0.03	−1.76	0.14
SDS	−0.03	0.07	−0.78	0.06
PDS	−0.15***	0.04	−3.75	0.31
Needs Punishment/Help	−0.25***	0.02	−6.61	0.53
	Support Toward SIFs			
Gender	−0.08*	0.08	−2.28	0.18
Age	0.14***	0.01	3.96	0.32
Race	0.03	0.09	0.73	0.06
Education	0.01	0.01	0.34	0.11
Political Ideology	0.25***	0.03	5.99	0.48
Religiosity	−0.07	0.04	−0.84	0.07
SDS	−0.11*	0.09	−2.57	0.21
PDS	−0.03	0.05	−1.84	0.15
Needs Punishment/Help	−0.18***	0.03	−4.53	0.37

Gender was dummy-coded (0 = men, 1 = women). Race was dummy-coded (0 = White, 1 = everyone else). Cohen’s *d* = 2 *t*/ √df. SDS = Social Distance Scale. PDS = Perceived Dangerousness Scale. Needs Help/Punishment = semantic differential question assessing participants’ view whether IDUs need help vs. punishment. *p < .05, **p < .01, ***p < .001

Regarding support toward SIFs (see Table 3), the regression model was significant and accounted for 24 % of the variance, *F* (9, 610) =21.27, *p* < .001. As predicted, more liberal political ideology, lower desire for distance from PWID, and stronger belief that they deserve help rather than punishment were all significantly related to more support. Older age also predicted greater support, and men reported stronger support for SIFs than women. Contrary to expectation, perceived dangerousness of PWID was not a significant predictor. Similarly, religiosity, race, and education were not significant predictors. Last, both political ideology and beliefs about PWID’s deservingness of punishment/help were the strongest predictors of support toward SIFs and had medium effect sizes. Age, gender, and desire for distance from PWID had small effect sizes.

## Discussion

The current study provided the first test in a U.S. sample of reported support for allocating government funds for both SIFs and NSPs if it could be shown they reduce harmful consequences of injection drug use. Results, which need to be considered in light of the non-representative sample characteristics, suggested that while the majority of the sample was at least “somewhat” supportive of both of these services, they were significantly more supportive of NSPs than SIFs, which may be partially related to the sample’s limited familiarity with SIFs. NSPs have become a symbol of harm reduction services for PWID [15], and despite the U.S. federal ban on harm reduction, numerous state and local governments have implemented NSPs [15]. Thus, it is possible that our participants have had greater exposure to NSPs compared to SIFs. This conjecture is consistent with findings that support for harm reduction tends to increase following implementation of those strategies [19, 37, 45]. However, there are also reports of increased public support towards SIFs over the course of six years despite of lack of implementation [36].

In line with extant literature [13, 36], while the majority of our sample supported SIFs and NSPs, the average endorsement scores were neither strongly favorable nor unfavorable, and there was considerable variability. Furthermore, we found that political ideology emerged as the strongest predictor of support toward both NSPs and SIFs, while age and gender played a smaller role. Consistent with the extant literature, more liberal political ideology was significantly related to higher support [13, 32]. Additionally, male gender and older age were significant predictors of higher support toward both NSPs and SIFs, but race and education were not. These results are consistent with some prior literature suggesting that male gender, but not race, is a significant predictor of support for drug policy initiatives [32]. However, there are contrasting prior findings related to gender [13, 39], education [13, 39] and age [39].

Clearly, we can only speculate about the reasons for the mixed findings, but some of the discrepant findings across studies might be due to methodological differences, such as the populations sampled (i.e., U.S. vs. Canadian adults). Also, our questions asked about attitudes towards providing government support for SIFs and NEPs vs. providing such services without specifying the funding source [13] or questions about general attitudes toward those services [39]. Hence, given the current study’s more direct policy focus, it is not altogether surprising that political ideology emerged as a stronger predictor than age or gender. In addition, it is possible that some individuals’ general attitudes towards harm reduction programs might differ from their specific attitudes towards providing government funding for these programs - an interesting question for future work.

Consistent with hypotheses, a stronger belief that PWID deserve help rather than punishment was related to more support toward both NSPs and SIFs. While preliminary and specific to this sample, these results are consistent with extant literature indicating relationships between different forms of public stigma and drug policy initiatives [13, 28, 32]. At the same time, lower perceptions of PWID as dangerous uniquely predicted more support toward NSPs, but desire for distance did not, while lower desire for distance from PWID uniquely predicted support toward SIFs, but perceived dangerousness did not. This differential prediction is both surprising and novel. To our knowledge, this is the first study to evaluate both perceived dangerousness and need for distance from PWID as unique predictors of support for various drug policy initiatives. One reason for the dissociation may simply be the overlap between the predictors (*r* = .53), which may have made it difficult to observe unique prediction by both variables when entered simultaneously in the model. This interpretation is supported by results of secondary analyses we conducted, indicating significant correlations between both stigma variables and support toward both NSPs and SIFs when examined independently. At the same time, we expect that perceived dangerousness and desired social distance will sometimes show variable relations with support for different types of drug policy initiatives, an important question to investigate in future research that evaluates a wider array of policies.

Although these results require replication, our findings related to public stigma have important implications for those with an interest in changing drug policy. Unlike demographic variables, public stigma can be shifted through invention efforts, and such interventions could be important not only for the general public but also for policy makers. For example, to the extent policy makers endorse these stigmatizing beliefs about PWID, it may partly explain the lack of federal support for harm reduction. This idea is consistent with the Resource Allocation Model [34], which suggests that perceptions of blame/responsibility directed towards those who need government resources are one factor that determines resource allocation decisions. This issue is not simply academic given robust evidence that individuals coping with substance abuse are viewed as personally responsible for their problems and deserving of punishment [7, 11, 12].

Thus, it may be important in education and intervention efforts to acknowledge and underscore the public stigma towards PWID (and individuals coping with substance use problems, more broadly). While we are not aware of any interventions targeting public stigma towards PWID, it may be possible to draw from the broader research on interventions designed to reduce stigma towards individuals with severe mental illness [6, 8, 9] and apply that to PWID. Specifically, there is support for both education (e.g., providing information about symptoms of mental illness or prevalence of different disorders) and contact strategies (e.g., making a personal connection between stigmatized individuals and members of the general public) in reducing stigma, with more robust support towards the latter [6, 9]. Our results, if replicated, raise the possibility that targeting perceptions of PWID as deserving help versus punishment could have a substantial impact on support toward strategies designed to help them, though this possibility needs to be tested using experimental rather than correlational methods. This may have impact at both the individual level, by reducing discrimination against PWID, and the policy level, by enabling a more evidence-based discussion about harm reduction strategies.

These results should be interpreted in light of several limitations. First, given that our sample was recruited online and the majority of participants were politically liberal, Caucasian adults, the generalizability of our data are limited. Second, our design is correlational, so it is not clear to what extent stigma predicts policy support or vice versa. Third, it would have been helpful to assess prior exposure to SIFs and NSPs, as well as personal history of substance use and/or of significant others’ history of substance use. These variables have been shown to be important predictors of public stigma, as well as support toward harm reduction for PWID [13, 28]. Fourth, the ‘double-barreled’ nature of the two policy items in our study (e.g., referencing both the transmission of HIV and discouraging the use of illegal drugs in the same item) can make interpretation of the observed support difficult. Hence, it is important that future studies include separate measures of support for NSPs to reduce HIV transmission and support for NSPs to discourage drug use. Finally, by adding the qualifier: “if it can be shown…” when connecting harm reduction strategies to scientific evidence, we addressed one of the gaps in the extant literature noted by Vernick and colleagues [40]. However, it is not clear how this addition influenced reported support, and raises questions about people’s knowledge about and valuing of scientific evidence to guide these policy decisions.

## Conclusion

Despite these limitations, the current study makes a number of novel contributions to the literature, including providing the first evaluation of U.S. support for SIFs. Results indicated the majority of participants support allocating government resources to fund harm reduction programs among PWID, and saw PWID as needing help rather than punishment. Further, beliefs that PWID deserve punishment, and are potentially dangerous and need to be avoided are strongly associated with less endorsement of harm reduction. Considering whether modifying these beliefs would shift support for harm reduction strategies will be an important next step, given the enormous personal and societal costs of injection drug use.
